# Determination of Luteolin and Apigenin in Herbal Teas by Online In-Tube Solid-Phase Microextraction Coupled with LC–MS/MS

**DOI:** 10.3390/foods13111687

**Published:** 2024-05-28

**Authors:** Atsushi Ishizaki, Akiko Miura, Hiroyuki Kataoka

**Affiliations:** School of Pharmacy, Shujitsu University, Nishigawara, Okayama 703-8516, Japan; ishizaki@shujitsu.ac.jp (A.I.);

**Keywords:** luteolin, apigenin, herbal teas, in-tube solid-phase microextraction, LC–MS/MS, online automated analysis

## Abstract

Herbal teas have attracted attention as functional beverages containing luteolin and apigenin, which exhibit antioxidant and anti-inflammatory effects. The objective of this study was to develop a sensitive online automated method to determine these flavones’ contents in herbal teas using in-tube solid-phase microextraction (IT-SPME) coupled with liquid chromatography–tandem mass spectrometry (LC–MS/MS). These compounds were extracted and concentrated by IT-SPME using a Supel Q PLOT capillary column and then separated and detected within 6 min using a CAPCELL PAK C18 MG III analytical column and a negative electrospray ionization-mode multiple-reaction monitoring system by LC–MS/MS. The detection limits (*S/N* = 3) for luteolin and apigenin were 0.4 and 0.8 pg mL^−1^, respectively, and the calibration curves were linear in the range of 2–2000 pg mL^−1^ with correlation coefficients above 0.9995, and intra-day and inter-day precisions with relative standard deviations below 2.9 and 3.6% (*n* = 6), respectively. The luteolin and apigenin in herbal tea were quantified using IT-SPME/LC-MS/MS following the acid hydrolysis of their glycosides. Among the 10 herbal teas tested, luteolin was detected in peppermint and sage at concentrations of 375 and 99 µg mL^−1^, respectively, while apigenin was detected in German chamomile at 110 µg mL^−1^, which were higher than in the other herbal teas. The method is expected to be a useful method for evaluating the efficacy of luteolin and apigenin in herbal teas as functional beverages.

## 1. Introduction

Flavonoids constitute a class of polyphenols characterized by a basic molecular structure comprising an aromatic A ring, a heterocyclic C ring, and an aromatic B ring interconnected by carbon-carbon bridges. They are categorized based on the position of hydroxy group bonds into subclasses such as flavones, flavonols, isoflavones, flavans, flavanols, flavanones, anthocyanidins, and others [1,2]. Flavonoids are secondary metabolites found in various plant sources including vegetables, fruits, tea leaves, and other natural products [3]. They play a role in plant self-defense mechanisms by absorbing UV light within the range of 280–315 nm to mitigate damage caused by reactive oxygen species [4]. Among flavonoids, flavones such as luteolin (3′,4′,5,7-tetrahydroxyflavone) and apigenin (4′,5,7-trihydroxyflavone), which have excellent pharmacological activities [5], induce the expression of antioxidant enzymes reducing oxidative damage and intracellular reactive oxygen species production [6,7,8,9,10]. Additionally, they show anti-inflammatory effects by suppressing the production of inflammation-inducing substances, such as nitric oxide and prostaglandin E2, and inhibiting cyclooxygenase-2 [6,7,11,12]; anticancer activities through cell cycle regulation and the induction of tumor cell apoptosis [6,13,14]; and antibacterial effects, directly killing bacteria and reducing bacterial pathogenicity [6,15,16]. Furthermore, these flavones are reported to be effective in preventing and treating gout by lowering uric acid levels through the inhibition of xanthine oxidase activity [17,18,19]. Luteolin and apigenin are found in vegetables such as parsley, spinach, peas, and celery [4,20,21,22,23,24] and fruits such as grapefruit, oranges, and olives [4,25], but since these flavones are not biosynthesized in humans, their potential as functional foods and supplements is increasingly recognized, particularly in herbal teas that contain significant quantities of flavones. It is important to clarify the content and consumption of these compounds in herbal teas from the perspective of disease prevention.

Luteolin and apigenin have been analyzed using high-performance liquid chromatography (HPLC) [20,21], LC–mass spectrometry (LC–MS) [24,26,27], LC–MS/MS [25,28,29], and high-performance thin-layer chromatography [30]. However, these methods have primarily focused on measuring their contents in the leaves, flowers, stems, and seeds of plants, with few reports on measuring their contents in herbal teas as beverages. Furthermore, these methods entail tedious and laborious pretreatments, such as solvent extraction, ultrasound-assisted extraction, and microwave-assisted extraction, to extract and concentrate these components from complex sample matrices, often necessitating the use of harmful organic solvents [20,21,22,23,24,25,26,27,28,29,30,31,32,33]. We previously developed a highly sensitive automated analytical method that utilizes in-tube solid-phase microextraction (IT-SPME) [34,35,36] coupled online with LC–MS/MS, demonstrating its effectiveness in analyzing various food samples [37,38,39]. The IT-SPME method employs an open-tube fused-silica capillary with a coated inner surface integrated into an LC autosampler for extraction, requiring minimal amounts of organic solvent and enabling automated analysis using online column-switching technology [36]. The development of efficient sample preparation and highly sensitive and accurate analytical techniques has been a challenge for the simple and rapid determination of luteolin and apigenin contents in herbal teas. The objective of this study was to establish an accurate and precise fully automated online analytical method for luteolin and apigenin in herbal teas using a modified IT-SPME LC-MS/MS/MS method, which further improves the extraction efficiency of the conventional IT-SPME method.

## 2. Materials and Methods

### 2.1. Reagents and Standard Solutions

**Figure 1:**
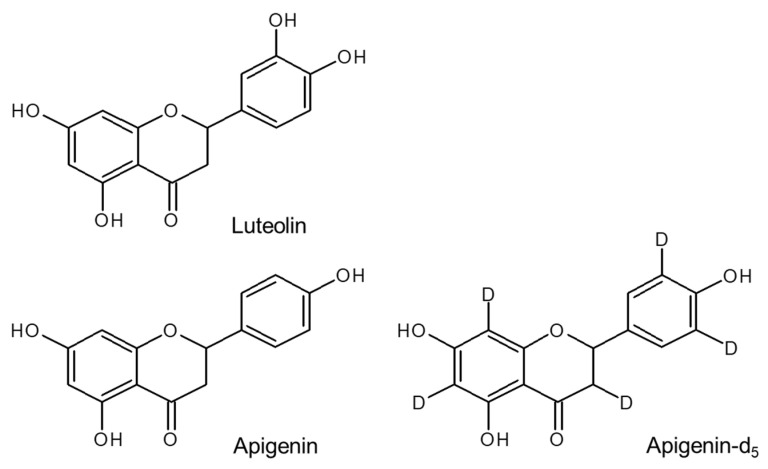
Structures of luteolin, apigenin, and apigenin-d_5_.

Luteolin and apigenin were procured from Tokyo Kasei Kogyo (Tokyo, Japan), while stable isotope-labeled apigenin-d_5_ (98% isotope purity) was obtained from Toronto Research Chemicals Inc. (TRC, North York, ON, Canada) and utilized as the internal standard (IS) (Figure 1). These standards and the IS were dissolved in LC–MS-grade methanol (Kanto Chemicals, Tokyo, Japan) to create a 1 mg mL^−1^ solution, securely capped and stably stored at 4 °C. Prior to use, working standard solutions were prepared by diluting these stock solutions with LC–MS-grade distilled water (Kanto Chemical) to obtain a concentration of 10 ng mL^−1^ each for luteolin and apigenin, and 1 ng mL^−1^ for the IS. LC–MS-grade acetonitrile (Kanto Chemicals), distilled water, and formic acid (Wako Pure Chemicals) served as mobile phases, while all other reagents were of analytical grade.

### 2.2. LC–MS/MS Analysis

LC-MS/MS analysis was performed using an online coupled LC-1260 Infinity and G6460C triple quadrupole mass spectrometer system (Agilent Technologies, Santa Clara, CA, USA). LC separation was performed utilizing a CAPCELL PAK C18 MG III column (100 mm × 2.0 mm I.D.; 5 µm particle size; SHISEIDO, Tokyo, Japan) maintained at a column temperature of 30 °C. The mobile phase consisted of a 0.1% formic acid aqueous solution/methanol with 0.1% formic acid (35/65, *v*/*v*) at a flow rate of 0.2 mL min^−1^ for 6 min. MS/MS analysis was performed in negative electrospray ionization (ESI^−^) multiple-reaction monitoring (MRM) mode. A nitrogen generator (Air Tech, Tokyo, Japan) was utilized for the nebulizer gas, with the following settings: a gas temp. of 300 °C, a flow rate of 10 L min^−1^, a nebulizer pressure of 40 psi, a sheath gas temp. of 400 °C, a sheath gas flow rate of 11 L min^−1^, and a capillary voltage of 3000 V. LC–MS/MS data analysis was conducted using Mass Hunter Workstation (Agilent Technologies). MRM transitions and setting parameters for luteolin, apigenin, and apigenin-d_5_ in Table 1.

### 2.3. In-Tube SPME

**Figure 2:**
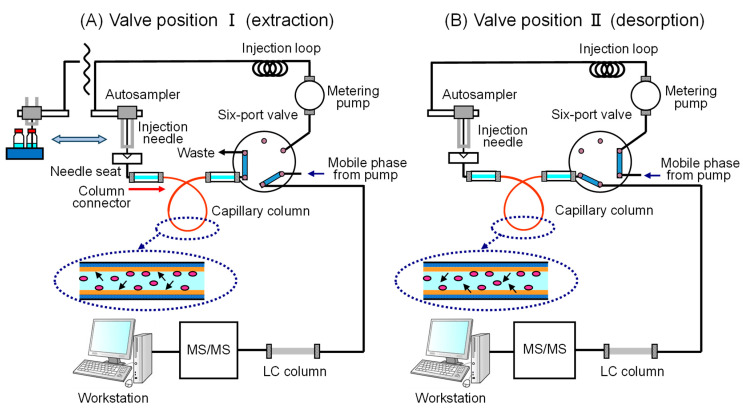
Schematic diagram of the improved IT-SPME LC-MS/MS system used in this study. (**A**) Adsorption of compound on stationary phase in capillary column, (**B**) desorption of compound by mobile phase solvent.

IT-SPME was performed by partially modifying the conventional draw/eject method [36]. As shown in Figure 2, in this setup, both ends of a GC capillary (60 cm × 0.32 mm i.d.) were threaded through a 2.5 cm sleeve of 1/16-inch polyetheretherketone tubing (330 µm i.d.), using standard 1/16-inch stainless-steel nuts, ferrules, and connectors. Instead of connecting between the injection loop and the needle, the setup was connected between the autosampler needle seal and the 6-way valve. Various extraction capillaries were employed to assess the extraction efficiency including Supel-Q PLOT (divinylbenzene polymer: 0.32 mm I.D., 12 µm: Supelco, Bellefonte, PA, USA), Carboxen 1006 PLOT (carbon molecular sieve: 0.32 mm I.D., 17 µm: Supelco), CP-Sil 5CB (100% polydimethylsiloxane; 0.32 mm I.D., 5.0 µm: Varian Inc., Lake Forest, CA, USA), CP-Sil 19CB (14% cyanopropyl-phenyl 86 polydimethylsiloxane; 0.32 mm I.D., 1.0 µm: Varian Inc.), and CP-WAX 52CB (polyethylene glycol; 0.32 mm I.D., 1.2 µm: Varian Inc.).

A 2 mL vial with a silicon/polytetrafluoroethylene (PTFE) septum was filled with the sample solution and placed in the autosampler. Additionally, vials containing 1.5 mL each of methanol and distilled water, as well as an empty vial, were placed in the autosampler. As shown in Figure 2A, according to the injection program, 40 µL of the solution was aspirated with a needle from the methanol and distilled water vials and injected into the capillary column for cleaning and conditioning. Subsequently, the compounds were adsorbed and concentrated in the stationary phase by repeatedly injecting 40 µL of the sample solution from the sample vial into the capillary column. Then, 50 µL of air was aspirated from the empty vial and discharged into the capillary column to ensure the complete drainage of the sample solution remaining in the capillary. Following needle washing, as shown in Figure 2B, a six-port valve was switched to enable the mobile phase to flow into the capillary column to desorb the compounds. These compounds were then separated by an LC column, with each compound detected by an MS/MS system, and the data were analyzed using a workstation. These series of IT-SPME controlled processes (Table S1) and the LC-MS/MS system were fully automated by Mass Hunter version B 07.00; Agilent Technologies, Santa Clara, CA, USA).

### 2.4. Method Validation Study

The linearity, limit of detection (LOD), limit of quantification (LOQ), precision, and accuracy of the developed analytical methods were validated following the criteria recommended by the ICH guidelines [40,41]. The linearity was assessed via three independent measurements at seven concentrations (2, 5, 10, 20, 100, 1000, and 2000 pg mL^−1^) of luteolin and apigenin in the presence of 0.1 mL of a 1 ng mL^−1^ IS solution. Calibration curves were constructed from the ratio of the peak height of each compound to the IS compound at each concentration. The LOD and LOQ were calculated from *S/N* ratios of 3 and 10, respectively, using a 1000 pg mL^−1^ solution. The intra-day (same day) and inter-day (six different days) precisions and accuracies were evaluated via six independent measurements at three concentrations (10, 100, and 1000 pg mL^−1^) each of luteolin and apigenin, expressed as relative standard deviations (RSDs, %).

### 2.5. Sampling and Preparation of Herbal Tea Samples

In this study, popular herbal teas that are commonly consumed were selected, and the 10 dried herbs examined in this study were purchased from Tree of Life Corporation (Gifu, Japan). The herbs (scientific name and country of origin) were *peppermint* (*Mentha × piperita* L., Egypt), *linden* (*Tilia europaea*, Bulgaria), *rosehips* (Rosa species, Chile), *lemongrass* (*Cymbopogon citratus*, Egypt), echinacea (*Echinacea purpurea* E., Germany), rosemary (Salvia Rosmarinus, Italy), *sage* (*Salvia officinalis*, Italy), *German chamomile* (*Matiricaria recutita* L., Egypt), elderflower (*Sambucus nigra*, Hungary), and *nettle* (*Urtica dioica*, Bulgaria). These herbs were grown in their respective countries of origin and processed for herbal teas, not harvested and processed from wild, native plants. These herbs were grown, processed, and then sold in polyethylene packages of 10–20 g each.

Following the general method for preparing herbal teas, 1.5 g of each dried herb was weighed into a screw-top Erlenmeyer flask with screw cap. Then, 150 mL of boiling water was added, the flask was capped, and the mixture was allowed to steep for 3 min. Next, 100 µL of each herbal tea obtained was measured into a 15 mL screw-capped test tube and mixed with 0.45 mL of 1 mol/L HCl. The tube was capped and heated to 100 °C for 90 min in a block heater (Thermo Fisher Scientific, Waltham, MA, USA). To neutralize the acid hydrolysis solution, 0.45 mL of a 1 mol L^−1^ sodium hydroxide solution was added. The mixture was then filtered through a hydrophilic PTFE membrane filter (pore size: 0.22 µm; Shimadzu GLC Ltd., Tokyo, Japan). Finally, 10 µL of the filtrate and 100 µL of a 1 ng mL^−1^ IS solution were measured into an autosampler vial, and the total volume was adjusted to 1 mL with water for IT-SPME LC–MS/MS analysis. The contents of luteolin and apigenin were determined as amounts per mL of herbal tea using a calibration curve.

### 2.6. Recovery Test of Luteolin and Apigenin Added to Herbal Tea Samples

The recovery test was conducted using rosemary herbal tea following the procedure in Section 2.5. Specifically, a 1 ng mL^−1^ IS solution and a standard mixed solution containing luteolin and apigenin were added to the herbal tea samples at concentrations of 10, 100, and 1000 ng mL^−1^, respectively, in the solution obtained after hydrolysis. Subsequently, the samples were analyzed using IT-SPME LC–MS/MS. The recovery rates of luteolin and apigenin were determined by calculating the difference between the concentration in the added sample and the concentration in the unadded sample, and then dividing this difference by the added concentration.

## 3. Results and Discussion

### 3.1. Optimization of IT-SPME of Luteolin and Apigenin

IT-SPME is a simple and widely used pretreatment method, facilitating the automated online extraction and concentration of target analytes from sample solutions without the use of organic solvents [34,35,36]. In conventional IT-SPME, the adsorption of compounds into the capillary column is achieved by repeatedly drawing/ejecting the sample solution into the capillary column. However, the compound concentration in the sample solution gradually decreases during this process, leading to a reduction in the extraction volume. In this study, to enhance the extraction efficiency, the capillary was positioned after the injection needle for unidirectional injection of the sample solution, as shown in Figure 2. Considering the contamination of the metering pump with the sample solution in flow-through IT-SPME [36], where a large amount of the sample is injected at once, a method involving the repetitive injection of a fixed volume of the sample solution was investigated. The length of the capillary is related to the amount of compound adsorbed, but an excessive capillary length prolongs the elution time and broadens the chromatogram peaks. Thus, a capillary length of 60 cm was used, with 40 µL of the sample solution repeatedly injected. This enhanced extraction efficiency at the same concentration of the solution can be repeatedly adsorbed in the capillary column. Also, the retention of the previous sample solution in the capillary until suction by the autosampler needle may improve the extraction efficiency.

**Figure 3:**
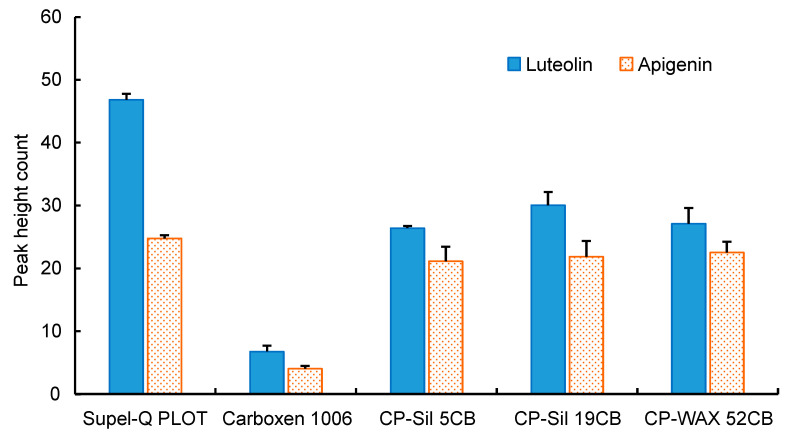
Effects of capillary coatings on IT-SPME of luteolin and apigenin. For each capillary column, 40 µL of a 100 pg mL^−1^ standard solution was repeatedly injected 5 times.

**Figure 4:**
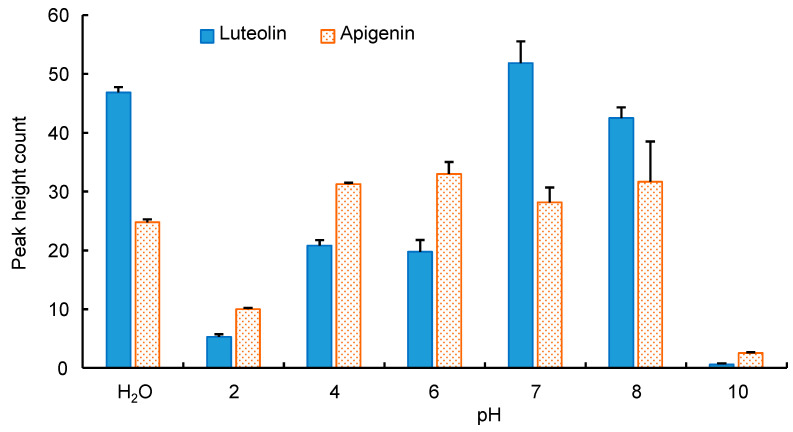
Effects of pH of sample solution on IT-SPME of luteolin and apigenin. For each pH, 40 µL of a 100 pg mL^−1^ standard solution was repeatedly injected 5 times.

**Figure 5:**
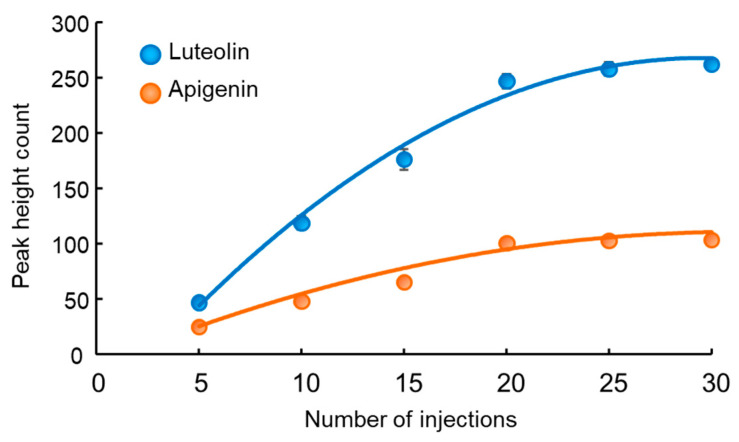
Effects of number of sample injections on IT-SPME of luteolin and apigenin. For each injection number, 40 µL of a 100 pg mL^−1^ standard solution was repeatedly injected.

To optimize the IT-SPME conditions, the effects of the capillary coatings, sample solution pH, and injection frequency of the sample solution were investigated using standard solutions containing 100 pg mL^−1^ each of luteolin and apigenin. Five commercially available capillary columns were assessed, among which Supel-Q PLOT, characterized by a porous adsorbent, showed the highest extraction performance due to its large adsorption surface area and thick film thickness (Figure 3). The pH of the sample solution was also examined using the Supel-Q PLOT capillary across a range of pH values (2, 4, 6, 7, 8, and 10). Although the highest extraction effect was obtained at around pH 7, no pH adjustment with buffer solutions was performed as a similar efficacy was achieved with distilled water (Figure 4). Furthermore, the injection frequency of 40 µL of the sample solution at a flow rate of 0.2 mL min^−1^ using a Supel-Q PLOT capillary was examined for 5, 10, 15, 20, 25, and 30 injections. While the extraction efficiency improved with the injection frequency, it reached a plateau, and 20 injections were deemed sufficient (Figure 5).

The luteolin and apigenin adsorbed in the capillary column were readily desorbed by the mobile-phase flow, and no carryover was observed. Upon comparison with the peak area counts obtained when 1 ng mL^−1^ luteolin and apigenin standard solutions were directly injected into the LC column, the absolute extraction rates into the capillary column under optimal IT-SPME conditions were calculated to be 42.9 and 39.5%, respectively. In contrast, when the same solution underwent the conventional IT-SPME method, the absolute extraction rates were calculated to be 5.8 and 7.2%, respectively. This indicates that the present improved method outperforms the conventional method.

### 3.2. LC–MS/MS Analysis of Luteolin and Apigenin

**Figure 6:**
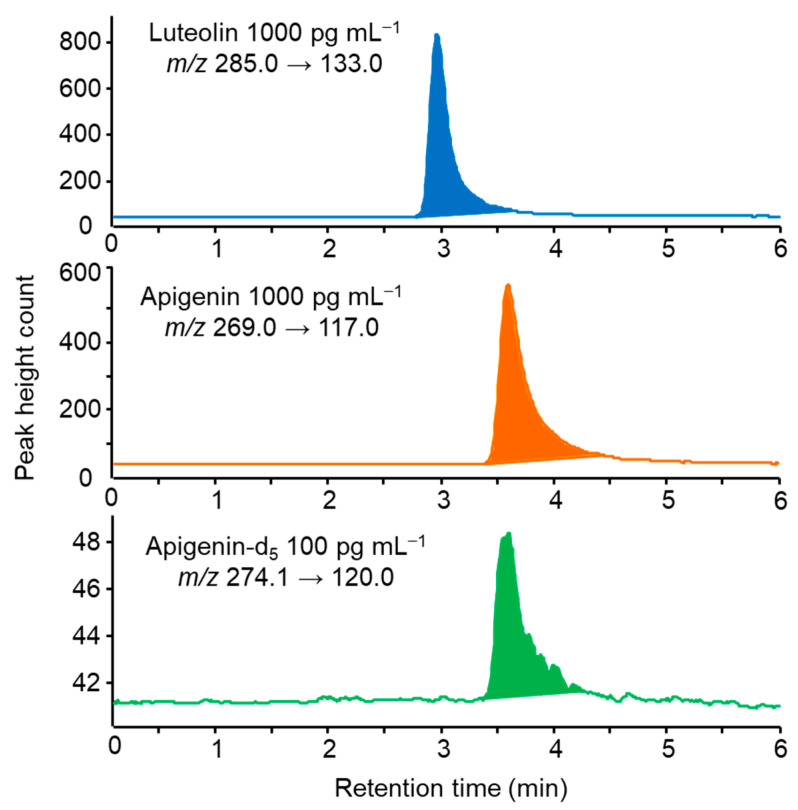
MRM chromatograms obtained from standard solution by IT-SPME LC-MS/MS in the negative ion mode. IT-SPME LC-MS/MS conditions are described in the Experimental Section.

Luteolin and apigenin, as well as apigenin-d5, showed good sensitivity in the ESI-negative ionization mode. The protonated ion [M–H]^−^ (Q1 mass) and the most intense fragment ion (Q3 mass) resulting from the cleavage of [M–H]^−^ for each compound were selected as precursor and product ions, respectively. The optimized MS/MS parameters and MRM transitions for each compound were in good agreement with previously reported results [32,33]. As shown in Figure 6, luteolin and apigenin could be separated and detected within 6 min using the CAPCELL PAK C18 MG III analytical column. However, during IT-SPME, the peaks exhibited slight tailing due to the spreading of the adsorption bands in the capillary column. With the developed online IT-SPME LC–MS/MS method, the analysis time per sample was about 20 min, enabling the automatic analysis of around 72 samples per day during nighttime operation.

### 3.3. Validation of the Developed Method

The performance of the developed IT-SPME LC–MS/MS method was validated by assessing analytical parameters, including linearity, LOD, and precision. As shown in Table 2, the calibration curves constructed from the peak height ratios of luteolin and apigenin to the IS showed a linear relationship within the range of 2 to 2000 pg mL^−1^ (seven points, with each concentration measured three times) with correlation coefficients exceeding 0.9995. The LODs (*S/N* = 3) of luteolin and apigenin were determined to be 0.4 and 0.8 pg mL^−1^, respectively, surpassing the sensitivity of the direct injection method (a 5 µL injection) by 73- and 79-fold, respectively. In addition, these results indicate that the proposed method is more than 150-fold more sensitive than previously reported methods [32,33]. The intra-day and inter-day precision (RSD, %) at low (10 pg mL^−1^), medium (100 pg mL^−1^), and high (1000 pg mL^−1^) concentrations were all within 5% for each compound, with accuracies ranging from 91 to 109% (Table 2). These results indicate that the present method largely meets the generally accepted validation criteria recommended by the ICH [40], enabling accurate and precise quantification.

### 3.4. Application to the Analysis of Herbal Tea Samples

Since many naturally occurring flavones like luteolin and apigenin exist as o-glycosides or c-glycosides of D-glucose, L-rhamnose, galactose, etc. [42,43], their glycosides were analyzed as aglycons via hydrolysis. In this study, due to the unavailability of standard glycosides of these compounds, sage herb tea was used to examine the hydrolysis reaction conditions. As shown in Figure S1, the amount of aglycones released increased with the rising temperature at a reaction time of 60 min, reaching almost equilibrium after more than 90 min at a reaction temp. of 100 °C, after which no degradation was observed. Hence, hydrolysis was conducted at a reaction temp. of 100 °C for 90 min. The recoveries of luteolin and apigenin added to the hydrolyzed samples ranged from 81.7 to 103.2% and 81.0 to 108.1%, respectively, falling within the acceptable ranges for quantitative analysis (Table 3). The LOQs (*S/N* = 10) for luteolin and apigenin were determined to be 1.2 and 2.6 ng mL^−1^ herbal tea, respectively, indicating that quantitative analysis could be performed with high sensitivity.

While herbal tea is usually prepared by adding boiling water to dried herbs and steeping for 2 to 3 min, it is believed that the levels of luteolin and apigenin in herbal tea may be influenced by both the temperature of the added hot water and the steeping duration. When the leaching amount of luteolin and apigenin was examined using sage, it was found that the leaching amount increased with rising temperature at a steeping time of 3 min and remained relatively constant at 100 °C for over 10 min (Figure S2). Since the steeping time affects the taste and aroma of herbal tea, it was prepared by adding 150 mL of boiling water to 1.5 g of the herb, steeping at 100 °C for 3 min, and using this as the sample for analysis in this study to determine the actual intake of luteolin and apigenin.

**Figure 7:**
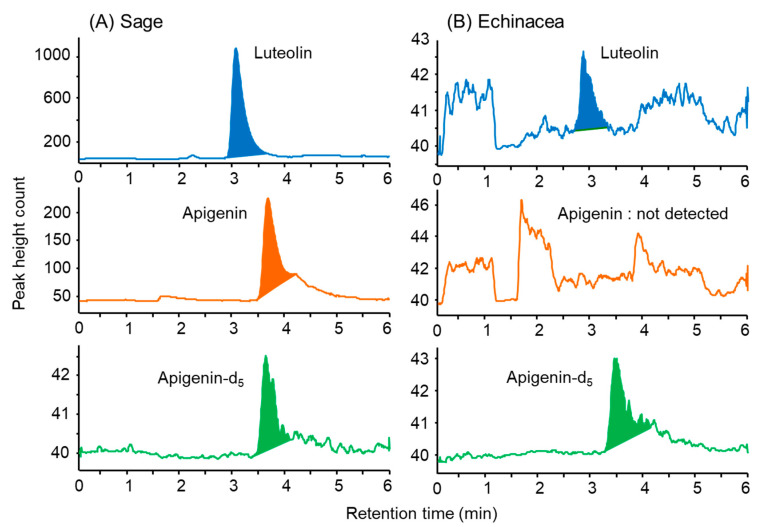
MRM chromatograms obtained from herbal tea by IT- SPME LC-MS/MS in the negative ion mode. IT-SPME LC-MS/MS conditions are described in the Experimental Section. MRM transitions are the same as in Figure 6.

As shown in Figure 7, luteolin and apigenin were detected as distinct and well-defined peaks. Using this method, the luteolin and apigenin contents in 10 herbal teas were determined. Luteolin was particularly abundant in peppermint and sage, containing 375 and 99 µg mL^−1^, respectively. Both are evergreen plants of the *Lamiaceae* family, and the dried herbs themselves are leaves. However, rosemary herbal tea did not have a higher content of luteolin, despite also being an evergreen plant in the *Lamiaceae* family. This might be attributed to the elongated leaves of rosemary having a smaller surface area and containing fewer leafy green components compared with other herbs. Conversely, the apigenin content was significantly higher in the German chamomile herbal tea, containing 110 µg mL^−1^ (Table 4). In a study on Japanese men, it was reported that daily intake of 10 mg of luteolin as a supplement alleviated gout inflammation due to its uric acid-lowering effect [44]. This study suggests that simply ingesting 150 to 200 mL of herbal tea containing peppermint, sage, or German chamomile once a day could be effective.

Herbal tea is globally popular as a therapeutic beverage utilized in various forms of traditional medicine. Its diverse health benefits and practical applications have been summarized [41]. In particular, peppermint and chamomile teas, among the most popular variants, and also rich in luteolin and apigenin contents, have been investigated for their biological activity and potential health benefits [45,46]. However, despite the wide availability of various herbal teas, the potential side effects of their consumption and health risks associated with the contamination of the plant material with toxic environmental contaminants remain unclear [41]. Thus, future research to investigate their clinical efficacy and evaluate the safety of herbal teas is warranted.

## 4. Conclusions

The IT-SPME LC-MS/MS method developed in this study was able to analyze luteolin and apigenin in small quantities of herbal tea with high sensitivity and selectivity, requiring minimal pretreatment beyond hydrolysis. This method is an environmentally friendly method that does not use organic solvents, and its online automated analysis allows unattended operation at night, saving labor costs. Since there are few reports on luteolin and apigenin contents in herbal teas, the results obtained in this study are new findings for evaluating the usefulness of herbal teas. This method is effective for accurately determining the luteolin and apigenin contents in herbal teas and is expected to be a useful tool in the development of functional foods aimed at preventing lifestyle-related diseases and promoting health through the utilization of medicinal herbs. Furthermore, the proposed analytical technique is expected to be expanded to the analysis of various bioactive compounds in other food samples, not only luteolin and apigenin.

## Figures and Tables

**Table 1:** MRM transitions and setting parameters for luteolin, apigenin, and apigenin-d_5_.

Compound	Retention Time (min)	Q1	Q3		FV^a^	CE^b^
			Quantifier	Qualifier		
Luteolin	3.0	285.0	133.0	150.9	170	37
Apigenin	3.6	269.0	117.0	151.0	130	41
Apigenin-d_5_	3.6	274.1	120.0	-	130	45

FV^a^: fragmentor voltage (V); CE^b^: collision energy.

**Table 2:** Linearity, sensitivity, precision, and accuracy of the IT-SPME LC–MS/MS method for luteolin and apigenin.

Compound	Linearity		LOD ^b^ (pg mL^−1^)		Concentration (pg mL^−1^)	Precision (RSD ^c^ %) (*n* = 6)		Accuracy (%) (*n* = 6)	
	Range (pg mL^−1^)	Linearity ^a^ (R^2^)	Direct Injection	IT-SPME		Intra-Day	Inter-Day	Intra-Day	Inter-Day
Luteolin	2–2000	0.9995	25	0.35	10	2.0	2.4	91.2	92.2
					100	2.3	3.5	94.3	96.1
					1000	1.1	2.8	95.0	96.9
Apigenin	2–2000	0.9999	63	0.79	10	2.2	2.5	106.9	107.5
					100	2.9	3.6	108.0	108.2
					1000	2.6	3.6	106.1	108.8

^a^ Correlation coefficient (*n* = 21). ^b^ Limits of detection (signal-to-noise ratio of 3). ^c^ RSD, relative standard deviation.

**Table 3:** Limits of quantification and recovery rates of luteolin and apigenin from herbal tea.

Compound	LOQ (ng mL^−1^)	Content (µg mL^−1^)		Recovery (%)
		Spiked	Mean ± SD (*n* = 3)	
Luteolin	1.2	0	0.2 ± 0.0	-
		10	8.4 ± 0.5	82
		100	86.1 ± 2.1	86
		1000	1032 ± 40	103
Apigenin	2.6	0	0.4 ± 0.0	-
		10	8.5 ± 0.5	81
		100	97.4 ± 2.7	97
		1000	1081 ± 71	108

**Table 4:** Contents of luteolin and apigenin in various herbal tea samples.

Herbal Tea	Content (µg mL^−1^)/Mean ± SD (*n* = 3)	
	Luteolin	Apigenin
Peppermint	375.4 ± 21.6	30.4 ± 1.7
Linden	3.5 ± 0.5	1.1 ± 0.1
Rosehip	0.1 ± 0.0	ND
Lemongrass	26.9 ± 1.0	0.6 ± 0.0
Echinacea	0.2 ± 0.0	ND
Rosemary	0.2 ± 0.0	0.4 ± 0.0
Sage	99.4 ± 2.7	37.0 ± 1.3
German chamomile	8.4 ± 0.9	110.0 ± 4.5
Elder flower	1.3 ± 0.2	1.4 ± 0.3
Nettle	0.9 ± 0.1	0.4 ± 0.1

## Data Availability

The data presented in this study are available upon request from the corresponding author.

## Supplementary Materials

The following supporting information can be downloaded at https://www.mdpi.com/article/10.3390/foods13111687/s1. Figure S1: Effects of reaction temperature and time on the release of luteolin and apigenin from sage herbal tea by acid hydrolysis; Figure S2: Effects of steeping temperature and time for leaching luteolin and apigenin from sage herbs into 150 mL of hot water; Table S1: Autosampler program for the IT-SPME control process in the autosampler software.
