# Supplementary material for: Determination of Luteolin and Apigenin in Herbal Teas by Online In-Tube Solid-Phase Microextraction Coupled with LC–MS/MS
Source: Foods. 2024 May 28;13(11):1687. doi: 10.3390/foods13111687 (PMC11172017; doi:10.3390/foods13111687)
Supplement: Supplementary file 1 [file foods-13-01687-s001.zip › foods-3025450-supplementary.pdf]

Supplementary Information

Determination of Luteolin and Apigenin in Herbal Teas by Online In-Tube Solid-phase Microextraction Coupled with LC–MS/MS

Atsushi Ishizaki [1], Akiko Miura [1] and Hiroyuki Kataoka [1,*]

[1] *School of Pharmacy, Shujitsu University, Nishigawara, Okayama 703-8516, Japan*

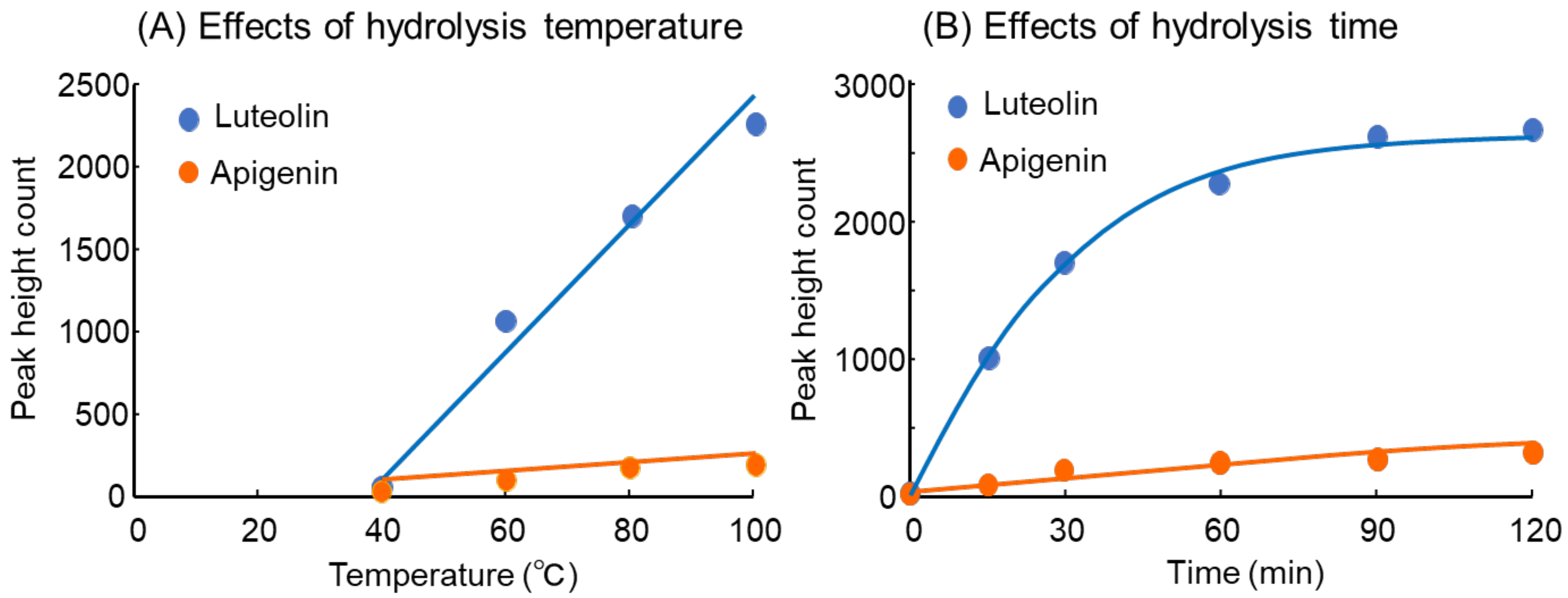

Figure S1. Effects of reaction temperature and time on the release of luteolin and apigenin from Sage herbal tea by acid hydrolysis. The hydrolysis process was carried out according to the operating procedure in Section 2.5 with (A) a constant reaction time of 60 min and (B) a constant reaction temperature of 100℃.

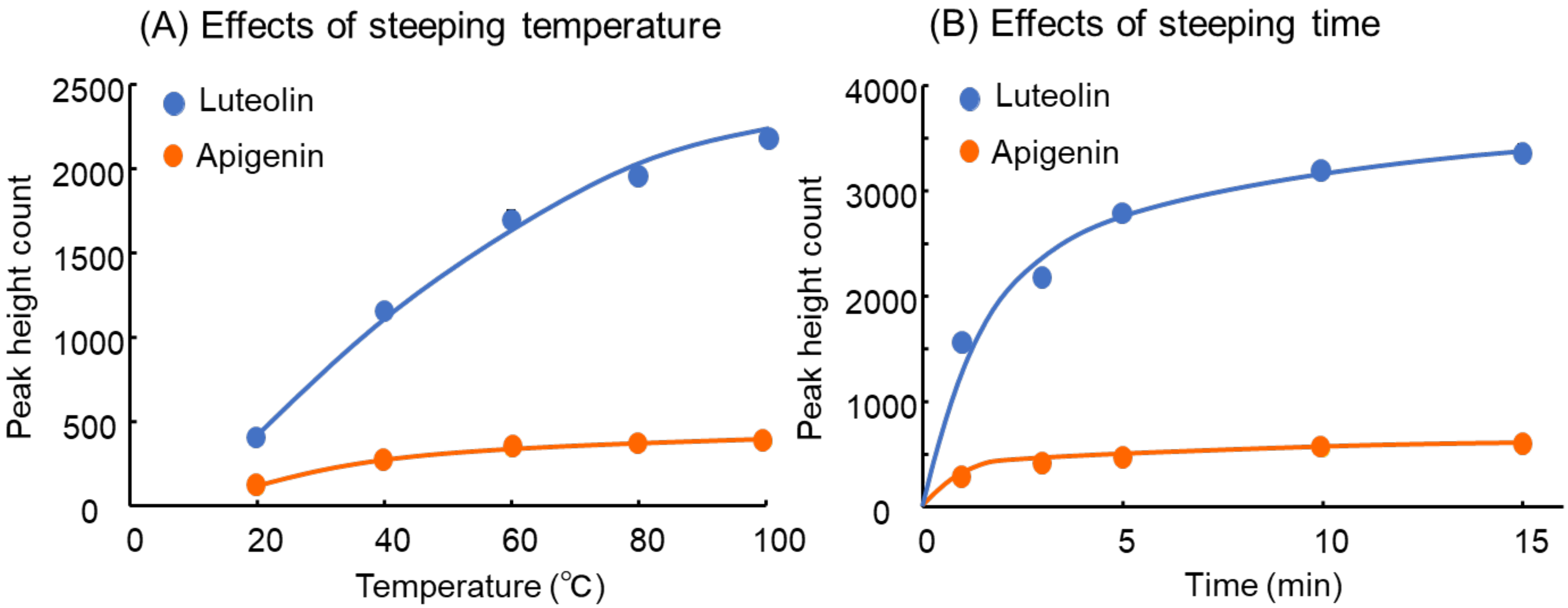

Figure S2. Effects of steeping temperature and time for leaching luteolin and apigenin from sage herbs into 150 mL of hot water. Herbal tea preparation was performed according to the operating procedure in Section 2.5 with (A) a constant steeping time of 3 min and (B) a constant steeping temperature of 100℃.

Table S1. Program for the IT-SPME process in the autosampler software.

Sequence	Event	Valve position	Vial position	Draw/ejection		
				Cycle	Volume (μL)	Speed (μL/min)
1	Conditioning of the capillary	I	MeOH	2	40	200
2	Conditioning of the capillary	I	Water	2	40	200
3	Extraction of analytes into the capillary coating	I	Sample	20	40	200
4	Drawing of air into the capillary	I	Empty	1	50	200
5	Needle washing	I	–	–	–	–
6	Injection	II	–	–	–	–
7	Desorption of analytes from the capillary coating by mobile phase flow	II	–	–	–	–
8	LC separation of analytes and return to sequence 1	I	–	–	–	–
